# Editorial: Sociocultural changes and adaptation: from mechanism to intervention

**DOI:** 10.3389/fpsyg.2023.1308078

**Published:** 2023-10-31

**Authors:** Yan Mu, Pan Liu, Alexander Scott English, Ren Li, Jieying Chen

**Affiliations:** ^1^CAS Key Laboratory of Behavioral Science, Institute of Psychology, Chinese Academy of Sciences, Beijing, China; ^2^Department of Psychology, University of Chinese Academy of Sciences, Beijing, China; ^3^School of Public Administration, Hunan University, Changsha, China; ^4^School of Psychology, Wenzhou-Kean University, Wenzhou, China; ^5^Department of Management and Marketing, The Hong Kong Polytechnic University, Hong Kong, China; ^6^Asper School of Business, University of Manitoba, Winnipeg, MB, Canada

**Keywords:** urbanization, sociocultural changes, tightness-looseness, adaptation, higher education, social norm

In the era of rapid urbanization, societies around the globe are experiencing profound shifts in societal and cultural dynamics. These transformations encompass diverse facets, from demographic changes to alterations in behavior patterns and psychological processes. Within this context, individuals are challenged to swiftly acquire new social norms, navigate cultural conflicts, and develop strategies for timely adaptation to evolving environments. The objective of this Research Topic was to understand the impact of these sociocultural changes on individuals and social groups. Within this Research Topic, Zhang et al. first introduced a three-stage social norm learning model, shedding light on the processes by which individuals adjust and adapt during sociocultural transformations. While gaining a deeper understanding of mechanisms at the individual level is valuable, Leng et al. validated the Cultural Tightness-Looseness Scale (CTLS) and General Tightness-Looseness Scale (GTLS), providing a reliable measure for cultures undergoing significant societal and cultural shifts, such as China. Furthermore, Lai et al. revisited the implications of urbanization in China, highlighting that higher education does not entirely postpone marriage, and the expansion of college enrollment can yield social and economic benefits. Finally, Zheng and Ishii examined the influence of distant and close social support on the psychological adaptation of Chinese international students, offering valuable insights into the complex interplay between cultural adaptation and social support in a population navigating urbanization.

## How we learn social norms: a three-stage model for social norm learning

As people from different regions or backgrounds migrate to urban areas seeking better opportunities, they may carry with them their own sets of social norms and practices. To quickly learn and adapt to social norms in urban settings is essential for social cohesion and successful adaptation to the urban environment. To fill this gap, Zhang et al. introduced a comprehensive three-stage social norm learning model, offering a roadmap for understanding how individuals adopt new social norms. It comprises three stages: pre-learning, where individuals gather initial social information; reinforcement learning, where they interact and adjust based on feedback; and internalization, where they voluntarily embrace the new norm. This model sheds light on the nuanced process of norm assimilation. Crucially, the authors emphasize the significance of internalization, as a pivotal facet of socialization.

In sum, the work of Zhang et al. not only elucidates the intricate dynamics of social norm learning but also sheds light on the critical role of internalization in the process. This work offers a comprehensive framework for comprehending the dynamics of individual adaptation and societal coordination amidst cultural transformations brought about by urbanization.

## Validation of the Chinese Cultural Tightness–Looseness Scale and General Tightness–Looseness Scale

Recently, cultural differences in the strength of social norms, often referred to as tightness-looseness (TL), have been widely observed (Gelfand et al., 2011; Chua et al., 2019). For example, Chua et al. (2019) identified TL discrepancies across the 31 Chinese provinces, with tighter norms prevalent in highly urbanized areas like Beijing, Shanghai, and Guangdong. These variations challenge the notion of a “universal” culture emerging with urbanization. Considering these findings, the Cultural Tightness-Looseness Scale (CTLS) developed by Gelfand et al. (2011), a tool for measuring perceived cultural tightness at a personal level, has gained widespread use. However, its reliability and suitability for specific cultures, such as China, remained uncertain.

To address this, Leng et al. validated the CTLS and GTLS on a large Chinese sample through a meticulous process. They used three samples: Sample 1 (*n* = 2,388) for item and exploratory factor analysis, Sample 2 (*n* = 2,385) for confirmatory factor and latent profile analysis, and Sample 3 (*n* = 512) for reliability and criterion validity, including a test-retest assessment with 162 participants after 4 weeks. Their efforts resulted in a revised CTLS with four items and a unidimensional structure, and a revised GTLS featuring eight items organized into Compliance with Norms and Social Sanctions dimensions. Latent profile analysis identified two distinct profiles for CTLS and GTLS scores, demonstrating the ability to categorize participants into high and low tightness perception groups. The revised Chinese versions exhibited strong criterion validity and reliable consistency, bolstering their utility.

This research not only validates the cultural tools for assessing tightness-looseness on Chinese populations but also underscores the importance of understanding how cultural tightness and looseness impact psychological wellbeing, particularly within the intricate tapestry of Chinese culture undergoing urbanization.

## Changes in behavior patterns or demographic structure? Re-estimating the impact of higher education on the average age of the first marriage

Urban environments, characterized by their diversity and multiculturalism, expose individuals to a wide spectrum of social norms and lifestyles. This exposure, intertwined with rapid urbanization, can influence marriage perspectives, potentially delaying it as people explore diverse aspects of life. Lai et al. studied the impact of higher education on marriage age using China General Social Survey data. Their findings reveal that higher education contributes to delaying first marriage age by 36.59% through behavior changes and 63.41% through demographic shifts. Regression analysis highlights a modest, yet discernible, effect of higher education on marriage age. Notably, demographic changes driven by higher education have a more substantial impact than behavioral shifts from the same expansion. In essence, higher education increases the average marriage age, primarily due to evolving demographics, delaying it by ~0.84 years.

The findings extend far beyond academia. They hold significant importance for policy considerations, particularly for developing countries like China, currently navigating the rapid waves of urbanization. In this context, the study addresses critical psychological consequences, such as evolving social preferences regarding marriage, and provides valuable insights for policymakers striving to foster positive societal adaptations in an era of urban transformation.

## Cross-cultural adaptation of Chinese international students: effects of distant and close support-seeking

Urbanization represents a significant demographic shift, often marked by the influx of individuals from diverse cultural backgrounds into burgeoning cities. For those leaving their homeland for urban life, understanding factors that ease their transition is crucial. Zheng and Ishii studied how distant and close support-seeking affects the psychological adaptation of Chinese international students in Japan and the United States. Their findings showed that distant support-seeking (from home country) negatively impacted adaptation, while close support-seeking (from host country) alleviated loneliness. Individuals with home-culture orientation sought distant support, while host-culture-oriented individuals preferred close support.

These insights into the dynamics of support-seeking behavior bear crucial implications for urbanization. Recognizing the pivotal role of emotional support, urban centers can implement targeted programs and initiatives designed to facilitate interactions and foster connections between newcomers and residents. Furthermore, acknowledging the nuanced preferences of individuals based on their acculturation orientation underscores the importance of personalized acculturation strategies. As cities continue to transform and diversify, these insights pave the way for more effective and empathetic strategies, ensuring that urbanization remains an enriching journey for all who embark upon it.

## Conclusion

In the wake of rapid urbanization, societies worldwide find themselves amidst a whirlwind of profound shifts in societal and cultural dynamics. The Research Topic focused on the impact of these sociocultural changes on individuals and social groups while delving into the mechanisms and factors underpinning this process. The four articles address this issue from different aspects: Zhang et al.'s model explains social norm acquisition, Leng et al. offer a validated tool for understanding cultural shifts, Lai et al., challenge common beliefs with policy implications, and Zheng and Ishii highlight the importance of social support in urbanization.

We sincerely thank all the contributors to this Research Topic for their valuable insights into the intricate relationship between urbanization, cultural adaptation, and societal transformation. Their efforts contribute to both academic advancement and the potential for policy improvements in a rapidly changing urban landscape.

## Author contributions

YM: Writing—original draft, Writing—review & editing. PL: Writing—review & editing. AE: Writing—review & editing. RL: Writing—review & editing. JC: Writing—review & editing.
